# One-Pot Method to Synthesize Silver Nanoparticle-Modified Bamboo-Based Carbon Aerogels for Formaldehyde Removal

**DOI:** 10.3390/polym14050860

**Published:** 2022-02-22

**Authors:** Wenxiang Jing, Chai Yang, Shuang Luo, Xiaoyan Lin, Min Tang, Renhong Zheng, Dongming Lian, Xuegang Luo

**Affiliations:** 1 Engineering Research Center of Biomass Materials, Ministry of Education, Southwest University of Science and Technology, Mianyang 621002, China; jwxzyy@163.com (W.J.); lxg@swust.edu.cn (X.L.); 2 Yibin Industrial Academy of Forestry and Bamboo, Yibin 644005, China; yang20062000@126.com (C.Y.); tang_min2121@163.com (M.T.); huashanbt0130@163.com (R.Z.); myue0130@163.com (D.L.); 3 Sichuan Tea College, Yibin University, Yibin 644000, China; luoshuang559520@163.com

**Keywords:** silver nanoparticle, bamboo-based carbon aerogel, adsorption, formaldehyde

## Abstract

The present study demonstrated a freeze-drying-carbonization method to synthesize silver nanoparticle-modified bamboo-based carbon aerogels to remove formaldehyde. The bamboo-based carbon aerogel (BCA) has the advantages of controllable pore size and rich oxygen-containing groups, which can provide a good foundation for surface modification. BCA can greatly enhance the purification of formaldehyde by loading silver nanoparticles. The maximum adsorption capacity of 5% Ag/BCA for formaldehyde reached 42 mg/g under 25 ppm formaldehyde concentration, which is 5.25 times more than that of BCA. The relevant data were fitted by the Langmuir model and the pseudo 2nd-order model and good results were obtained, indicating that chemical absorption occurred between the carbonyl of formaldehyde and the hydroxyl of BCA. Therefore, silver nanoparticle-modified bamboo-based carbon aerogels play a positive role in the selective removal of formaldehyde. Silver nanoparticles promoted the activation of oxygen and strengthened the effect of BCA on HCHO adsorption.

## 1. Introduction

Formaldehyde (HCHO) is a colorless, pungent, and irritating gas. It is a highly active aldehyde and an important chemical raw material, and so many industrial products cannot be produced without it. Due to natural and man-made processes, it can be seen everywhere indoors and outdoors [1]. Indoor HCHO is mainly produced by wood products, thermal insulation materials, paint, varnish, household cleaning products and cigarettes, etc. [2]. Formaldehyde is toxic and one of the main harmful volatile gases. Therefore, it is identified as a carcinogenic and teratogenic substance [3,4]. It was reported that animals developed headaches, dizziness, nausea, and even vomiting when exposed to a low concentration of formaldehyde for a long time [5,6]. Michal Krzyzanowski [7] reported that children in high formaldehyde environments and tobacco environments had a significantly higher probability of developing asthma and chronic bronchitis compared to those in low concentration environments. Therefore, it is necessary to explore an effective method to purify formaldehyde. There are many kinds of purification methods; according to the purification principle, these mainly include: plant purification [8,9,10], adsorption [11,12], photocatalysis [13,14,15], and so on. 

The nano silver catalyst has excellent catalytic activity and reaction selectivity due to its small particle size, high specific surface area and many surface-active points, and the bond state and coordination of surface atoms are very different from those in particles. It can be used as a catalyst for a variety of oxidation reactions, such as NO_x_ elimination [16,17,18], CO oxidation [19,20,21], ethylene epoxidation [22,23,24], and methane oxidation [25,26]. The main factors affecting the catalytic performance of silver are the type and properties of support, loading amount, loading mode, nanoparticle size, morphology, pre-treatment atmosphere, and temperature. 

Compared with other precious metals, silver has nontoxic characteristics, is cost-effective, and has high applicability for air purification. The oxygen in the air and organics are adsorbed and oxidized on the surface of silver nanoparticles. At room temperature, molecular oxygen decomposes into oxygen atoms or oxygen ions with high oxidizing ability on the surface of silver nanoparticles [27]. This conforms to the oxidation mechanism of organic compounds on silver nanoparticles. The oxygen atoms or molecules adsorbed on the surface of silver particles can contribute or receive electrons, leading to the generation of active oxygen, and then oxidize organic compounds in the air.

Carbon aerogel is one of the best materials for a catalyst carrier due to its high specific surface area, high porosity, low density, and good stability. Meanwhile, because bamboo has the characteristic of a high content of cellulose (40–50%) [28], bamboo cellulose was used as a raw material to synthesize the precursors of silver-loaded cellulose gel, and then a freeze-drying-carbonization method was used to synthesize carbon aerogels with controllable porosity and purity. In order to evaluate its effect on formaldehyde removal, untreated bamboo-based carbon aerogels and silver nanoparticle-modified carbon aerogels were evaluated by XRD, XPS, SEM-EDS, TEM, a surface area and porosity analyzer, as well as for adsorption capacity and breakthrough time.

## 2. Materials and Methods

### 2.1. Materials

Bamboo pulp was purchased from Sichuan Tianzhu Bamboo Resources Development Co., Ltd, Yibin, China. Silver nitrate (AR, ≥99%) was purchased from ChengDu Chron Chemicals Co., Ltd, Chengdu, China. N-methyl morpholine-N-oxide, NMMO (AR, ≥99%), was purchased from Bide Pharmatech Ltd, Shanghai, China. Formaldehyde solution (AR, 37~40%) was purchased from Chengdu Jinshan Chemicals Co., Ltd, Chengdu, China. Ultra-pure water was self-made, resistivity ≥18.25 MΩ·cm. 

### 2.2. Preparation of Bamboo-Based Cellulose Carbon Aerogel (BCA)

Bamboo-based cellulose (0.5 g) and 20 g NMMO were put into a beaker, and 3.04 mL of the different aqueous silver nitrate solutions (0 mg/mL, 1.645 mg/mL, 4.934 mg/mL, and 8.224 mg/mL) were added. The mixture was stirred and heated for 2 h at 90 °C; the pre-gel was cooled to room temperature overnight. Cellulose hydrogels were regenerated with deionized water to remove the NMMO. Subsequently, these hydrogels were pre-frozen (−24 °C) and freeze-dried (temperature of −60 °C, vacuum of 0.06 MPa) for 48 h. During the carbonization process, these aerogels were heated at 350 °C for 4 h with a heating rate of 10 °C/min and argon atmosphere of 0.1 L/m in a furnace. The samples were named BCA, 1%Ag/BCA, 3%Ag/BCA, and 5%Ag/BCA, respectively. The experiment was replicated three times.

### 2.3. Characterization

The morphology analysis of the samples was tested by SEM (Quanta FEG 250, FEI, Ann Arbor, MI, USA) and TEM (FEI Tecnai G2 F20, Columbus, OH, USA). The specific surface area/porosity of the samples was analyzed by a BET surface area measurement (ASAP2020, Micromeritics, Norcross, GA, USA). The BET model was used to analyze the specific surface area of the aerogel at 77.35 K, the DFT model was used to analyze the total porosity, and the HK model was used to analyze the microporosity. The surface chemical properties and composition were analyzed by XPS (Escalab 250Xi ThermoFisher, Waltham, MA, USA). The chemical composites on the aerogel surface were analyzed by XRD (D8 Advance, Bruker, Billerica, MA, USA).

### 2.4. Breakthrough Curves’ Measurement by HCHO

**Figure 1:**
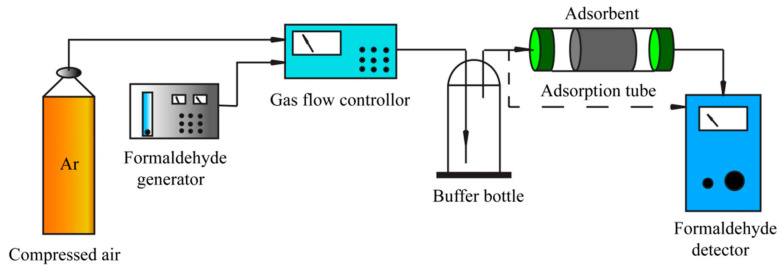
Schematic diagram of breakthrough curve measurement.

The dynamic adsorption of HCHO on samples (Figure 1) was measured by breakthrough curves. One gram of BCA was put into a U tube reactor, and the adsorption temperature was 25 °C by water bath. The concentration of HCHO in the system was kept stable (approximate 25 ppm, 50 ppm, and 70 ppm) by adjusting the argon flow. Meanwhile, the concentration of HCHO was detected online by an electrochemical sensing gas detector (MIC-600-4, Erantex) [29]. The experiment was replicated three times.

### 2.5. Adsorption Capacity of HCHO

**Figure 2:**
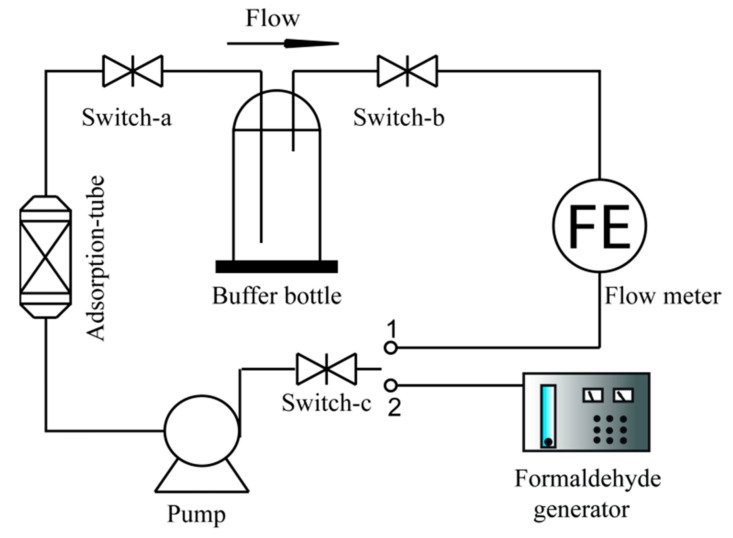
Schematic diagram of HCHO adsorption capacity measurement.

The HCHO adsorption capacity was determined by a homemade device (Figure 2). First, in order to make a certain concentration of HCHO fill the system, switch-c was connected with position 1, while the formaldehyde generator was connected to the system. Second, the adsorption tube containing a certain amount of sample was connected into the system, the pump was turned on to make a certain concentration of HCHO circulate within the system, the adsorption tube was weighed at regular intervals, and the adsorption capacity (q_t_) was calculated using Equation (1). The experiment was replicated three times.

(1) $$q_{t}=\frac{m_{1}-m_{2}}{m_{0}}$$

where *m*_1_ is the weight of the adsorption tube after adsorption, g, *m*_2_ is the weight of adsorption tube before adsorption, g, and *m*_0_ is weight of BCA, g.

## 3. Results and Discussion

### 3.1. Characterization of Carbon Aerogels

**Figure 3:**
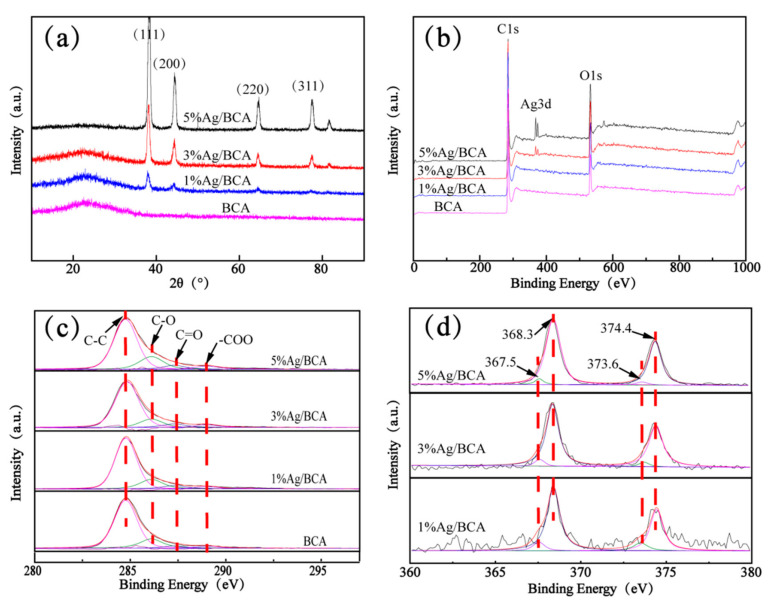
(**a**) XRD pattern, (**b**) XPS survey, (**c**) C1s, and (**d**) Ag3d of silver nanoparticle-modified BCAs and untreated BCA.

The carbon aerogels with different Ag contents were studied by XRD and XPS (shown in Figure 3). Figure 3a shows the XRD spectra of BCA and Ag/BCA with different capacities of silver nanoparticles. BCA and Ag/BCAs at the 2θ = 23° diffraction peak were graphitic (002) plan, indicating that BCA and Ag/BCA were composed of graphite-like microcrystalites. The diffraction peaks of (111), (200), (220), and (311) corresponding to Ag of the Ag/BCA at 38.1°, 44.3°, 64.7° and 77.5° indicated that nano silver particles were loaded on BCA. Since the diffraction peaks of (111) and (200) were stronger than the conventional value, this indicated that Ag2O was present in the experiment [30,31].

Figure 3b shows the XPS survey of BCA and Ag/BCAs. The spectra revealed that carbon (284 eV) and oxygen (532 eV) were the major elements, which might be due to the large amount of hydroxyl and aldehyde groups in cellulose as precursors. At the same time, the results showed that the peaks of Ag3d (368 eV) were stronger with the increase in AgNO_3_, which indicated that more silver nanoparticles were successfully loaded in BCAs. Figure 3c shows the C1s spectra of three silver nanoparticle-modified BCAs (Ag/BCAs) and untreated BCA. The same four peaks of all samples are shown, which could be attributed to graphitic and aromatic groups (C-C) at 284.8 eV, a hydroxyl group (C-O) at 286.1 eV, an aldehyde group (C=O) at 287.3 eV, and a carboxyl group (-COO) at 289.1 eV, respectively [32,33]. Figure 3d shows the Ag3d spectra of three Ag/BCAs with different silver nanoparticles contents. The characteristic peaks of Ag3d were 368.3 eV and 374.4 eV, which were due to the two-spin orbit coupling, but the peaks of 367.5 eV and 373.6 eV implied the existence of Ag-O [30,31].

**Figure 4:**
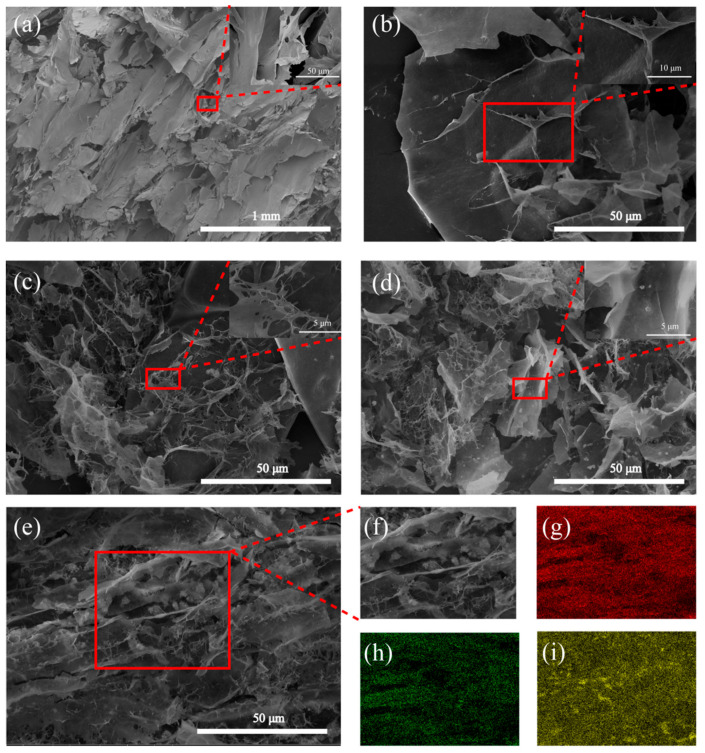
SEM of (**a**) bamboo-based cellulose aerogel, (**b**) BCA, (**c**) 1% Ag/BCA, (**d**) 3% Ag/BCA, and (**e**,**f**) 5% Ag/BCA. EDS elemental mapping of (**g**) carbon (red), (**h**) oxygen (green), and (**i**) silver (yellow) of 5% Ag/BCA.

**Figure 5:**
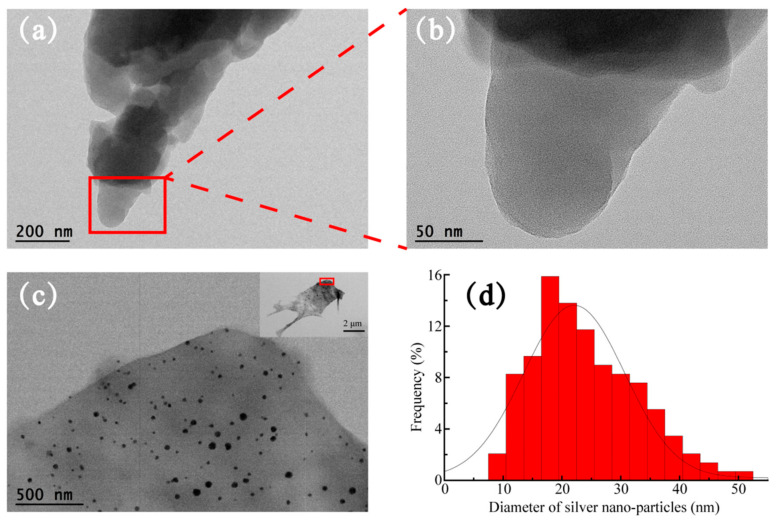
TEM of (**a**,**b**) BCA and (**c**) 5% Ag/BCA; (**d**) size distribution of silver nanoparticles.

The surface ultrastructure, main element type, and distribution of BACs and Ag/BACs were analyzed by SEM-EDS and TEM, as shown in Figure 4 and Figure 5. Figure 4a shows that the cellulose aerogels exhibited a three-dimensional structure and smooth surface [34,35], and the carbonized aerogels still maintained a smooth three-dimensional structure, as shown in Figure 4b. Figure 4c–e shows aerogels with different silver nanoparticles contents; it can be clearly seen that Ag/BACs had more pores than BAC. The reason for this phenomenon was that AgNO_3_ was heated to produce gas, which then caused the pores in the BCAs. According to Figure 4f–i, the carbon, oxygen, and silver in 5% Ag/BCA were evenly distributed [36].

The morphologies of BAC and 5% Ag/BCA were analyzed by transmission electron microscopy (see Figure 5). Through the comparison of BAC (Figure 5a,b) and 5%Ag/BCA (Figure 5c), it can be seen that the silver particles in the 5%Ag/BCA were evenly distributed in the shape of spots. In total, 140 silver nanoparticles were selected from 5% Ag/BCA for analysis (Figure 5d). The average size of the silver nanoparticles was 25.42 nm.

**Figure 6:**
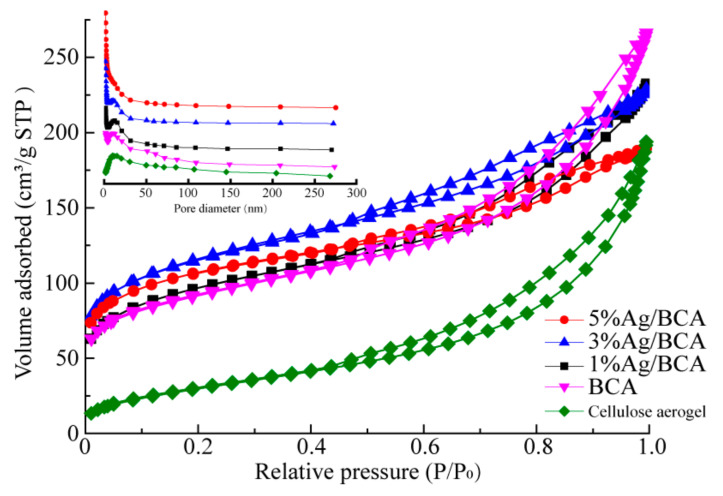
N_2_ adsorption/desorption isotherm and pore size distribution (insert).

Figure 6 shows the N_2_ adsorption/desorption isothermal curves and pore size distribution (insert) of cellulose aerogel, BCAs, and Ag/BCAs. According to IUPAC classification specifications, the N_2_ adsorption/desorption isotherm curves of all the samples belong to type Ⅰ with H3 hysteresis loops, implying the existence of slit pores formed by sheet particle packing [37,38]. The specific surface area (S_BET_), average pore size (D_pore_), and pore volume (V_pore_) derived from the isotherms are listed in Table 1. The precursors of all samples showed an increase in specific surface area during carbonization, especially for Ag/BCAs. Therefore, the results indicated that carbonization was a pore-forming process. However, with higher silver loading, the pore volume of the Ag/BCAs showed a decreasing trend, indicating that part of the pores was occupied by silver nanoparticles.

### 3.2. Adsorption Performance

#### 3.2.1. Adsorption Process

**Figure 7:**
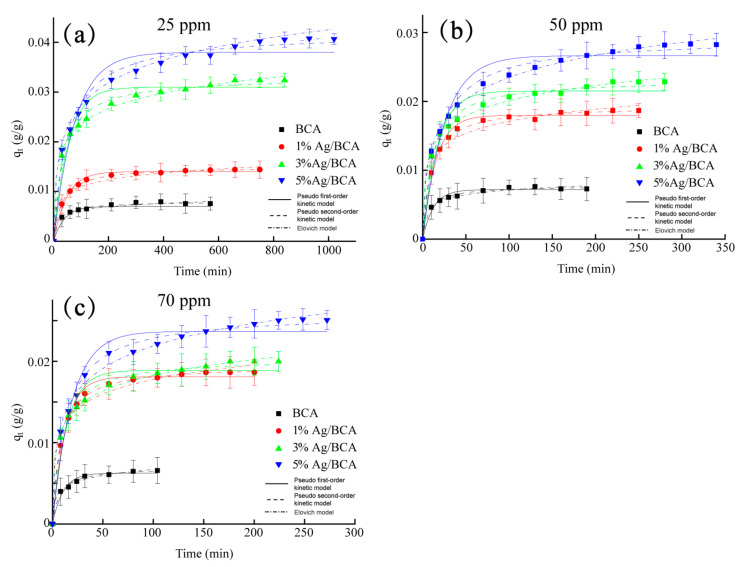
Adsorption kinetics for the different concentration of HCHO (**a**) 25 ppm, (**b**) 50 ppm, (**c**) 70 ppm.

Figure 7 showed the absorption of BCAs and Ag/BCAs to different concentration of HCHO. All the samples were able to adsorb the formaldehyde. However, the silver nanoparticle-modified BCAs obviously had a higher effect on the absorption of HCHO. In the lower concentration of the HCHO (25 ppm), the maximum adsorption capacity of 5% Ag/BCA reached 42 mg/g (Figure 7a), and the absorption capacity was improved with the higher amount of silver nanoparticles. In the other concentrations (50 ppm and 70 ppm), the same results were found (Figure 7b,c). Meanwhile, for the same adsorbent, the formaldehyde absorption capacity showed a downward trend with the higher concentration of HCHO, which was also consistent with previous reports [39,40]. This phenomenon might be caused by the different instantaneous flow rate through the adsorbent. Additionally, the adsorption process of all samples was investigated, the relevant data were fitted by the pseudo 1st order kinetic model, the pseudo 2nd order kinetic model, and the Elovich model; the results are shown in Table 2. The results showed that the adsorption processes of the samples fit better the pseudo 2nd-order kinetic model (R^2^ = 0.991~0.999) than the pseudo 1st-order kinetic model (R^2^ = 0.966~0.988) and Elovich model (R^2^ = 0.975~0.997).

#### 3.2.2. Adsorption Thermodynamics

**Figure 8:**
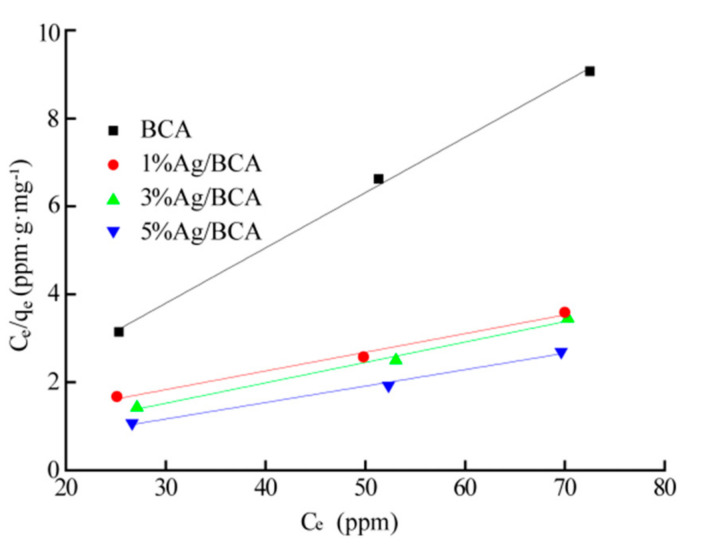
Langmuir isothermal model.

Figure 8 shows the Langmuir model of all the samples, and the fitting results are shown in Table 3. The effect of BCAs and Ag/BCAs on the HCHO absorption could be described by the Langmuir isothermal model (R^2^ > 0.98), indicating that chemisorption occurred between the carbonyl groups of formaldehyde and the hydroxyl of the BCAs [41,42]. The qmax of Ag/BCAs was higher than that of BCA, indicating that the silver nanoparticles had a certain positive effect on formaldehyde removal.

#### 3.2.3. Breakthrough Curves

**Figure 9:**
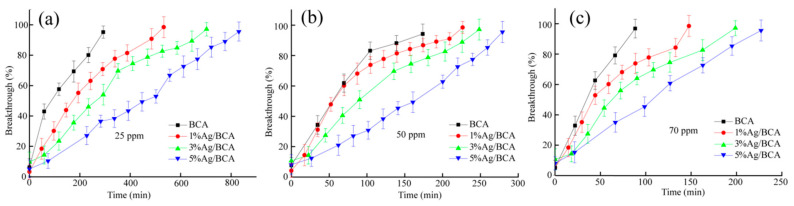
Breakthrough curves for the different concentrations of HCHO gas (**a**) 25 ppm, (**b**) 50 ppm, (**c**) 70 ppm.

In order to explore the dynamic adsorption process of BCAs and Ag/BCAs, the breakthrough curves at different formaldehyde concentrations (25 ppm, 50 ppm, and 70 ppm) are shown in Figure 9. The breakthrough times of BCA, 1% Ag/BCA, 3% Ag/BCA, and 5% Ag/BCA decreased from 293 to 88, 533 to 147, 702 to 199, and 831 to 227 min, respectively. Moreover, the higher the formaldehyde concentration at the inlet, the shorter the breakthrough time. The phenomenon may be attributed to the fact that Ag/NO_3_ produced a large amount of gas to enlarge the specific surface area during the pyrolysis of precursors. In addition, the presence of silver nanoparticles also provided some new binding sites for formaldehyde adsorption [43].

#### 3.2.4. Comparison of Adsorption Performance of Different Ag Loaded Materials

Compared with the other Ag-loaded materials in the literature (Table 4), the Ag/BCAs had a better ability to remove HCHO. The reason may be caused by different synthesis methods and the different initial concentration of formaldehyde. In addition, Ag/BCA had potential advantages for gas treatment applications. On the one hand, the process of synthesis was very environmentally friendly, and no harmful reagent was used. On the other hand, the efficiency was high. The absorption capacity of Ag/BCA was higher than that of Ag/ZnO-5.

#### 3.2.5. Mechanism Analysis of HCHO Adsorption on Ag/BCAs

**Figure 10:**
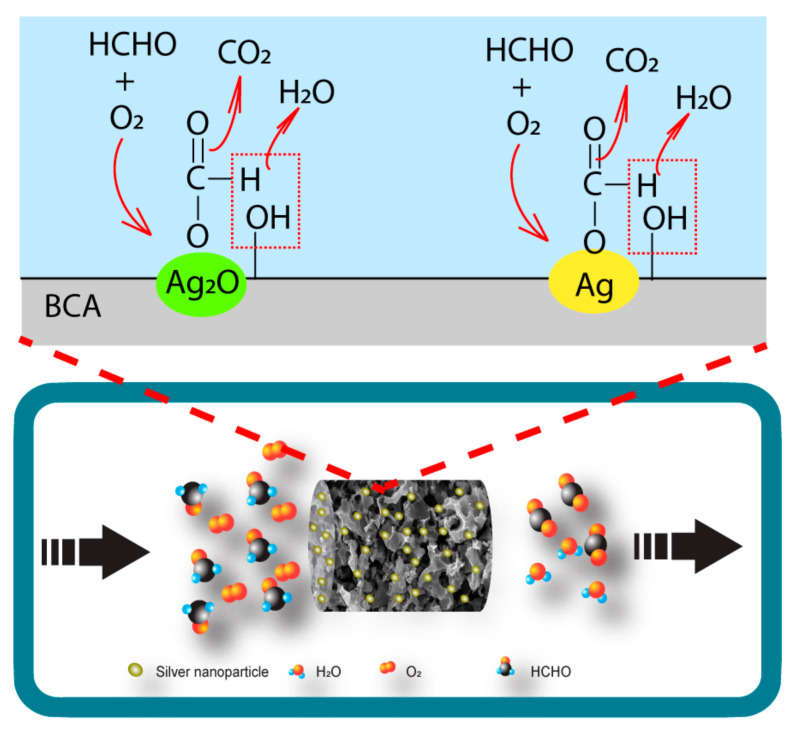
Possible mechanism of HCHO adsorption on Ag/BCAs.

Based on the above experimental results of the adsorption process and breakthrough curves, the possible mechanism of Ag/BCAs adsorbing HCHO is proposed by Figure 10. The process included three main steps: (i) Formaldehyde was adsorbed on the surface of Ag/BCAs; meanwhile, O_2_ and H_2_O formed a series of oxygen groups (-OH, O^−^ and O_2_^−^) on the surface of the silver particles. (ii) The adsorbed HCHO was oxidized to formate or carbonate. This reaction was the rate-limiting step of the whole oxidized process. (iii) The intermediates were further decomposed into CO_2_ and H_2_O [48,49].

## 4. Conclusions

In summary, the silver nanoparticle aerogel precursors were synthesized by a simple one-pot method and freeze-dried using bamboo cellulose as the raw material; then, the silver nanoparticle carbon aerogel was obtained after carbonization. The performance of the modified carbon aerogels was measured by modern instruments, such as XRD, XPS, SEM-EDS, TEM, and a surface area and porosity analyzer. The large number of oxygen-containing functional groups on the surface of the silver-loaded carbon aerogels provided the binding sites for the silver nanoparticles, which provided favorable conditions for the removal of formaldehyde. Meanwhile, a large amount of gas was generated from AgNO_3_ decomposition during carbonization, pore structures became more abundant, and the specific surface area and pore volume were improved, which also provided new binding sites for the adsorption of formaldehyde. The kinetic models of formaldehyde adsorption by the Ag-loaded carbon aerogels fit the pseudo second order kinetic model, and at a certain concentration of formaldehyde, the adsorption capacity of the Ag-loaded carbon aerogel increased with the increase in Ag loading. Thermodynamic data could be well represented by a Langmuir model, which indicated that the chemisorption might be occurring between the carbonyl groups of formaldehyde and the hydroxyls of BCA; meanwhile, silver-loaded carbon aerogels had a positive effect on the selective removal of formaldehyde. Silver nanoparticles might have synergistic effects with BCA on HCHO adsorption. The existence of silver nanoparticles promoted the activation of oxygen, which contributed to the oxidation of HCHO.

## Figures and Tables

**Table 1:** Physical properties of as-synthesized samples.

Sample	S_BET_ (m^2^/g)	D_pore_ (nm)	V_pore_ (cm^3^/g)
Cellulose aerogel	111.27	10.15	0.28
BCA	324.99	4.92	0.40
1% Ag/BCA	329.97	4.25	0.35
3% Ag/BCA	359.29	3.24	0.35
5% Ag/BCA	394.20	3.54	0.29

**Table 2:** Kinetic model parameters for HCHO adsorption on the BCAs and Ag/BCAs.

Model	Parameter	BCA			1%Ag/BCA			3%Ag/BCA			5%Ag/BCA		
		25 ppm	50 ppm	70 ppm	25 ppm	50 ppm	70 ppm	25 ppm	50 ppm	70 ppm	25 ppm	50 ppm	70 ppm
Pseudo 1st order kinetic	K1 (min^−1^)	0.026	0.080	0.095	0.021	0.065	0.080	0.019	0.058	0.072	0.013	0.040	0.052
	qe (g/g)	0.007	0.007	0.006	0.014	0.018	0.018	0.031	0.022	0.019	0.038	0.027	0.024
	R^2^	0.966	0.966	0.968	0.988	0.987	0.988	0.951	0.950	0.951	0.943	0.944	0.954
Pseudo 2nd order kinetic	K2 (g/g·min)	5.555	17.31	20.27	2.274	5.407	6.503	0.915	3.932	5.531	0.459	1.956	2.925
	qe (g/g)	0.008	0.008	0.007	0.015	0.019	0.020	0.033	0.023	0.020	0.042	0.029	0.026
	R^2^	0.991	0.991	0.991	0.999	0.997	0.999	0.991	0.991	0.992	0.997	0.998	0.990
Elovich	α (g/g·min)	0.005	0.016	0.006	0.005	0.022	0.025	0.008	0.018	0.018	0.004	0.007	0.009
	β (g/g)	998.9	1037	938.7	503.8	395.2	386.3	220.2	314.0	356.0	150.6	215.4	248.9
	R^2^	0.984	0.983	0.992	0.976	0.975	0.977	0.996	0.996	0.996	0.997	0.997	0.992

**Table 3:** Langmuir isothermal model parameters for HCHO adsorption.

Adsorbent	Langmuir Model Parameters		
	q_max_ (mg/g)	K_L_	R^2^
BCA	7.953	4.783	0.997
1% Ag/BCA	21.56	0.0757	0.983
3% Ag/BCA	23.56	0.3504	0.987
5% Ag/BCA	26.75	0.8314	0.987

**Table 4:** Comparison of Ag/BCAs with other Ag-loaded materials.

Adsorbent	Adsorbent Type	S_BET_ (m^2^/g)	V_pore_(cm^3^/g)	Initial HCHO Concentration (ppm)	Adsorption Capacity of HCHO (mg/g)	Ref.
Ag-AC	Activated carbon	685	-	349.9	0.51	[43]
0.001 M Ag-AC	Activated carbon	1145	0.66	0.5	0.47	[39]
HKUST-1	MOF	1733	0.89	0.164	0.50	[44]
2.5 wt%-Ag NPs@ZIF-8	Zeolite	1190	0.64	1.41	2.27	[45]
Ag/ZnO-5	ZnO	8	0.12	10	12.76	[46]
Ag-Na/CeO_2_-N	CeO_2_	92	0.17	970	0.200	[47]
Ag/BCA	Aerogel	394	0.29	25	42.00	This work

## Data Availability

The data presented in this study are available on request from the corresponding author.
